# ASPHER Statement: Towards a Carbon-Neutral Association of Schools of Public Health in the European Region

**DOI:** 10.3389/phrs.2021.1604127

**Published:** 2021-05-06

**Authors:** Rana Orhan, John Middleton, Katarzyna Czabanowska

**Affiliations:** ^1^ Association of Schools of Public Health in the European Region (ASPHER), Brussels, Belgium; ^2^ Department of International Health, Care and Public Health Research Institute CAPHRI, Faculty of Health, Medicine and Life Sciences, Maastricht University, Maastricht, Netherlands; ^3^ Department of Health Policy Management, Institute of Public Health, Faculty of Health Sciences, Jagiellonian University, Krakow, Poland

**Keywords:** climate change, climate action, public health association, social responsibility, corporate sustainability

“Climate change is the biggest global health threat of the 21st century” was The Lancet’s conclusion in 2009 [1]. Yet, public health institutions are insufficiently acting on climate change mitigation.

Any increase in global warming leads to negative impacts on human health and well-being [2]. In 2017, the European Union accounted for 7.78% of global greenhouse gas emissions, which is the driver of human-induced climate change [3]. To reach the European Commission’s 2030 reduction target of at least 50%, an upsurge of climate actions is needed across all sectors [2, 4]. As Zotova et al. stated in the context of the medical community, “unprecedented, ambitious action, and innovative thinking are urgently needed” [5].

Action is also highly needed in public health associations, which have a corporate responsibility towards society. Their actions should be best practices in public health and specifically climate mitigation. One of these associations, the Association of Schools of Public Health in the European Region (ASPHER), is concerned with their current behaviour and their contribution to climate change. With the COVID-19 pandemic causing many conferences to be postponed or moved to an online version, we have the momentum to change climate-damaging behaviour.

**FIGURE 1:**
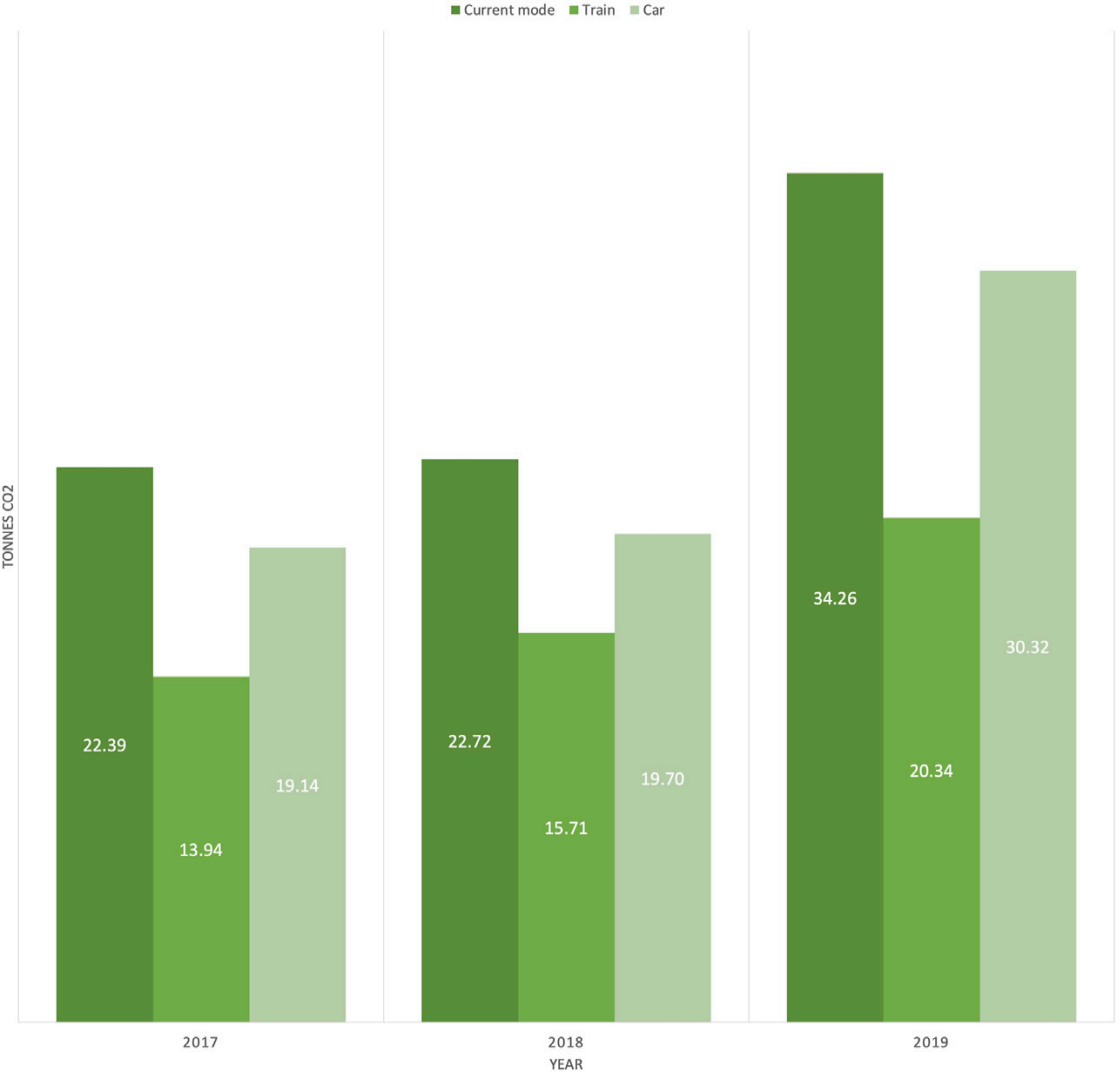
Break-down of carbon footprint (tonnes CO_2_) by current mode and alternatives by ASPHER Executive Board and Secretariat members in 2017, 2018 and 2019.

We estimated the carbon footprint of the ASPHER Executive Board (EB) and Secretariat in the past three years (2017–2019). These representatives travelled for the biannual EB meetings, the annual ASPHER members’ meeting (“Deans’ and Directors’ Retreat”) and the European Public Health (EPH) Conference. We estimated that the total footprint was 79.37 tonnes CO_2_; with 34.26 tonnes in 2019, 22.72 tonnes in 2018 and 22.39 tonnes in 2017 (Figure 1). This equals 17.1 passenger vehicles driven for one year. The actual carbon footprint is much higher since carbon footprint from in-city travelling, accommodation, food waste and other carbon-producing behaviours were not calculated. Moreover, it is only a fraction of the cost of ASPHER members’ travel, average participation at Deans’ and Directors’ Retreats being around a hundred attendees.

Travelling by train would have reduced ASPHER’s carbon footprint by 40.62% in 2019, 30.84% in 2018, and 37.76% in 2017. By car, this would have been 11.51, 13.30, and 14.51%, respectively. By rail, ASPHER could have reduced its carbon footprint of the past three years by 29.38 tonnes (37.01%) which is above the average of 26.46 tonnes produced by the current mode of travel. By car, this would have been 12.87% (10.21 tonnes).

These results show a worrying trend in the carbon footprint. We argue that the association needs to invest in stimulating their representatives towards climate change mitigating behaviour. First, we suggest replacing the standalone EB meetings in Brussels with virtual conferencing; there was already interest in moving towards virtual conferencing [6]. Second, we advise combining face-to-face meetings with other meetings, which is good behaviour already shown by ASPHER in the past. Third, we recommend choosing meeting locations that are more readily accessible by train, hereby acknowledging that an alternative to air travel is not always an option as rails and roads are often suboptimal. Our calculations show a significant difference in carbon footprint between two meetings in the same year: travelling to the Deans’ and Directors’ Retreat in Erice (Italy) produced 11.51 tonnes CO_2_, while the EPH Conference in Marseille (France) is only accountable for 3.21 tonnes. Another consideration would be the numbers of representatives required to attend international meetings on ASPHER’s behalf. Furthermore, we recommend carbon offsetting to compensate for emissions that cannot be avoided [7]. Only 13 out of 191 reported travels have been carbon offset, leaving 71.51 tonnes CO_2_ not offset. We urge that ASPHER compensates their carbon footprint from the past years’ travels, equating to €1,859. Carbon offsetting should be included in ASPHER’s policy as standard for future travels and by future event organisers for all attendees. Last, ASPHER should implement targets for its actions, in keeping with the European Union’s 2030 target. We believe ASPHER can exceed this target in a much shorter timescale: we recommend a 70% reduction in 3 years. Indeed, we anticipate that many of the behavioural changes enforced by the coronavirus will remain as part of the “new normal” after the pandemic.

This analysis of ASPHER’s travel behaviour shows that there is still a lot to gain by public health professionals and their associations. The public health field has a contributing role to the detrimental impact of human behaviour on the climate and therefore human health, and holds a social responsibility towards society. Our recommendations mean minor changes to an association’s activities; however, they will have a great positive impact on the climate.
